# Robust machine learning modeling for multi-parameter prediction in friction stir welding of naval brass: a case study towards industry 4.0

**DOI:** 10.1038/s41598-026-49005-0

**Published:** 2026-05-22

**Authors:** Adeel Shehzad, Syed Farhan Raza, Adeel Ikram, Ahmed Murtaza Mehdi, Muhammad Umar Farooq, Mehdi Tlija

**Affiliations:** 1 Automotive Engineering Centre, University of Engineering and Technology, G.T. Road, Lahore, 54890 Pakistan; 2 Department of Industrial and Manufacturing Engineering, University of Engineering and Technology, G.T. Road, Lahore, 54890 Pakistan; 3 Smart Manufacturing and Renewable Technologies Laboratory (SMART-Lab), Department of Mechanical Engineering, The Superior University, Lahore, Pakistan; 4 Faculty of Medicine, The University of Queensland, Frazer Institute, 37 Kent Street, Woolloongabba, 4102 Australia; 5 Department of Mechanical Engineering, University of Michigan, Ann Arbor, MI USA; 6 Industrial Engineering Department, College of Engineering, King Saud University, P.O. Box 800, Riyadh, 11421 Saudi Arabia

**Keywords:** Friction stir welding, Predictive modelling, Data augmentation, Industry 4.0, Engineering, Materials science, Mathematics and computing

## Abstract

This study establishes a comprehensive machine learning (ML) framework for accurately predicting essential friction stir welding (FSW) attributes in naval brass, focusing on weld temperature, strength, and hardness. To address insufficient experimental data, a customized variational autoencoder (VAE) was used to generate high-fidelity augmented datasets. The fidelity of the augmented datasets was verified through distributional similarity analysis and feature-correlation divergence measures, ensuring the synthetic samples strictly adhered to the underlying physical-statistical signatures of the experimental data. A comparative assessment of four ML algorithms, support vector regression (SVR), AdaBoost, XGBoost, and decision tree (DT), demonstrated that SVR consistently attained greater predictive accuracy. The SVR model produced mean absolute percentage errors (MAPEs) of 1.0% for weld temperature, 3.2% for weld strength, and 1.6% for weld hardness. The model’s generalization was further validated with an independent experimental dataset, affirming its reliability for practical industrial applications. The results demonstrate that ML models can effectively elucidate intricate process-property correlations in friction stir welding of naval brass, reducing reliance on experiments and enabling data-driven optimization. The suggested framework facilitates intelligent process monitoring, quality control, and the incorporation of Industry 4.0 methods in advanced welding applications.

**Supplementary Information:**

The online version contains supplementary material available at 10.1038/s41598-026-49005-0.

## Introduction

Friction stir welding is a solid-state joining technology that may be assisted with various aids to improve joint quality. These technological aids include vibrations and water, converting FSW into friction stir vibration welding (FSVW) and underwater FSW (UWFSW). While extensive research exists on FSW, FSVW, and UWFSW, most studies focus on materials other than brass^1–13^.

Brass is an alloy of copper and zinc, and its properties can be controlled by varying the percentages of both alloying elements. Brass has numerous excellent properties, such as brilliant strength, excellent corrosion resistance, and the best electrical and thermal conductivities. The tensile strength of CuZn30 brass is 360 MPa^14^. Industrial applications of brass alloy are extensively found in piping, heat exchangers, cartridge casings, radiator cores, door handles, valve guides, builder’s hardware, and electrical terminals. On the other hand, FSW is being widely applied in the automotive, marine, and construction industries, where brass alloys are considered exceptional candidates. However, fusion welding of brass is problematic, which is usually accomplished at the melting point of brass. When joining various alloys, e.g., brass, welds of improved strength with minimum brittleness are typically desired. Furthermore, FSW is more appropriate for materials, e.g., brass, as it joins below their melting points.

Therefore, brass may easily be welded using solid-state joining methods, omitting fusion welding processes. Visually, the FSW joint maintained a consistent aesthetic quality, appearing free from significant macroscopic defects both before and after welding. As fusion welding processes involve melting brass, evaporating zinc, and leaving porosity issues in the welded brass^15,16^. Conclusively, solid-state joining (e.g., FSW) studies for brass material are scarce. Joining brass alloys in their solid state is critically important to avoid reaching the melting point, thereby preventing zinc evaporation. To address this requirement, FSW presents a viable solution, as it is a solid-state joining technique that eliminates the need for melting. While few studies have investigated FSW for different brass alloys, this technique presents considerable promise for producing high-quality welds while minimizing zinc loss^14,17–20^. In addition to the benefits offered by FSW, the performance of the joint can be further improved with suitable post-weld treatments. One study^21^ indicated that electric pulse treatment on linear friction-welded TC17/TC4 joints significantly improves ductility by refining the microstructure and diminishing microstructural gradients.

Machine learning models are widely employed for prediction, optimization, and decision support across a broad range of research areas^22–25^. For FSW applications, Industry 4.0 tools enable real-time process monitoring, dynamic adjustment of welding parameters, and early identification of potential defects^26^. Within this context, ML approaches have gained increasing attention as data-driven techniques for modeling and predicting critical weld responses. For example, Mothilal and Kumar^27^ applied ML-based regression models to predict the weld strength of dissimilar aluminum alloys, including AA7075 (Al–Zn) and AA5083 (Al–Mg). The ML models were trained by conducting 20 experiments obtained using various FSW parameters. Among the ML models, the decision tree showed outstanding performance, with an R^2^ score of 0.97. Anandan and Manikandan^28^ welded the dissimilar aluminum (Al) alloys, namely 7050 and 2014 A. The authors used ML regression models to monitor and predict peak temperature in the heat-affected zone (HAZ). Mechanical properties of the welded joint are strongly susceptible to temperatures above 350 °C during FSW of dissimilar Al alloys. Among various regression models, random forest regression (RFR) was appropriate for predicting the peak temperature during FSW. A Mishra^29^ applied the ML models on FSW of various magnesium (Mg) alloys, including AM20, AZ61A, AZ31B, and AZ31. XGBoost achieved higher performance, with an R^2^ score of 0.81, compared to other ML models. Fuse et al.^30^ designed an ML framework to predict the tensile strength of Al alloys welded by FSW. Among the ML algorithms tested in their work, the adaptive boosting classifier (ABC) was found to be the most accurate, achieving 81.6% accuracy. Chen^31^ presented an ML approach integrating the Bayesian theorem to forecast residual stress, plastic deformation, and peak temperature. Training data was collected using extensive numerical simulations based on input FSW. The regression analysis showed excellent predictive performance with coefficients of determination of 0.969, 0.955, and 0.919, respectively. Some other researchers have also used ML models to predict the FSW performance for Al alloys^32–34^.

A fundamental prerequisite for all ML applications is the availability of model training data, a demand characterized by a dual nature: the imperative for representative data that accurately reflects the target function, and the demand for a substantial number of data observations^35^. Although the use of AI can improve manufacturing processes in many areas, such as productivity, quality assurance, and so on, its application is typically hindered by the limited amount of data^36^. One of the commonly used approaches to overcome the scarcity of manufacturing process data is data augmentation. However, data augmentation has been widely used in computer vision, pattern recognition, and image processing. Despite the potential benefits of data augmentation, its implementation with numerical data in the context of manufacturing processes is markedly restricted and encounters significant challenges^37^. Moreover, performing extensive experimentation in manufacturing is a cumbersome process that may incur significant cost and time. Given these challenges in manufacturing data scarcity and the limitations of conventional augmentation methods for numerical data, this study develops a variational autoencoder-based approach to augment the data effectively. The proposed method addresses the dual requirements of data quality and quantity, enabling more robust training of subsequent ML models in manufacturing applications.

**Table 1:** Comparative summary of ML techniques employed in FSW.

Ref	FSW material	Techniques	Data augmentation	Evaluation metrics			Targeted weld parameters		
				MAPE	RMSE	MAE	Temp	Strength	Hardness
^27^	Al-Zn and Al-Mg	DT, RF, XGBoost, CatBoost, AdaBoost	ⅹ	ⅹ	ü	ü	ⅹ	ⅹ	ⅹ
^31^	Al alloy	Bayesian ML, SVM, CNN, RF	ⅹ	ⅹ	ⅹ	ⅹ	ü	ⅹ	ⅹ
^38^	Mg alloy	DT, XGBoost, ANN, RF, GB, AdaBoost	ⅹ	ⅹ	ⅹ	ⅹ	ⅹ	ü	ⅹ
^39^	Cu	KNN, DT, DT (Information grain)	ⅹ	ⅹ	ⅹ	ⅹ	ⅹ	﻿ü	ⅹ
^40^	AA6082	RF, M5P Tree Regression, ANN	ⅹ	ⅹ	ü	ü	ⅹ	ü	ⅹ
^41^	6061AA	DT, RF, GB	ⅹ	ⅹ	ⅹ	ü	ⅹ	ü	ⅹ
**Ours**	**Naval Brass**	**SVR**,** AdaBoost**,** XGBoost**,** DT**	**ü**	**ü**	**ü**	**ü**	**ü**	**ü**	**ü**

While the existing body of literature indicates some progress in the friction stir welding of brass alloys and acknowledges the inherent difficulties posed by fusion welding, a significant gap in this domain is the absence of any documented predictive FSW model for brass alloys using ML. As shown in Table 1, existing ML applications in FSW predominantly focus on aluminum alloys, with limited studies on copper and a notable lack of comprehensive multi-parameter predictions that address weld temperature, weld strength, and weld hardness simultaneously, as undertaken in this study. Moreover, the FSW of naval brass 405 − 20 was accomplished for the first time by the authors of this manuscript, with the exception of our prior publication^42^. No prior research has explored the use of ML on naval brass specifically to enable Industry 4.0 applications, such as advanced process monitoring and control. This work’s unique contribution is its method of data augmentation using a customized variational autoencoder (VAE), trained solely on experimental data from naval brass friction stir welding (FSW). The enhanced datasets underwent thorough validation to ensure reliability and representativeness, addressing the issue of insufficient experimental data and enabling robust predictive modeling of the three essential weld parameters. This study together provides (i) efficient data augmentation by VAE, (ii) thorough dataset validation, (iii) multi-parameter predictive modeling, and (iv) robust generalization for industrial FSW process monitoring, facilitating the implementation of Industry 4.0 practices.

## Materials and methods

**Fig. 1:**
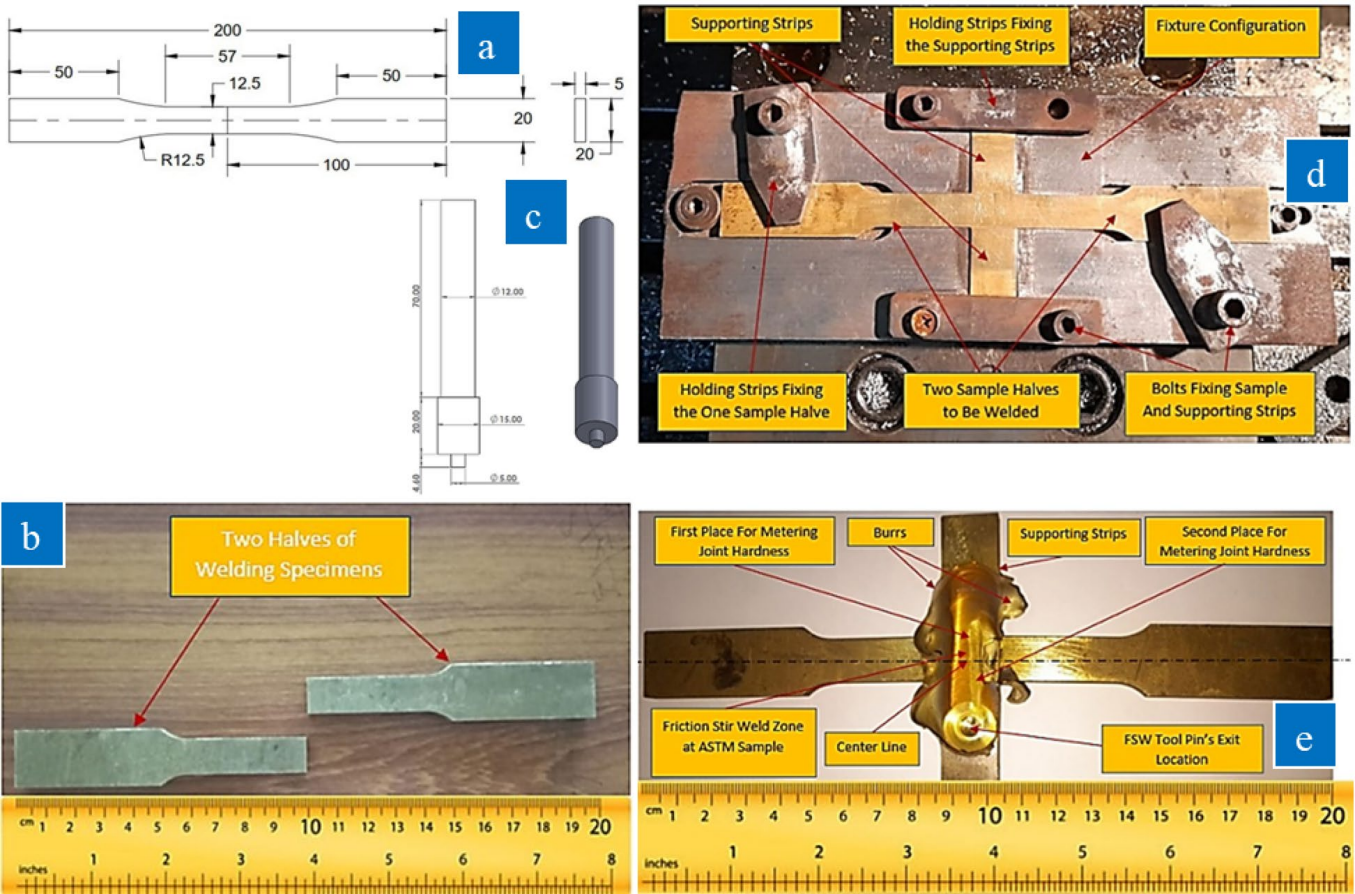
(**a**) Specimen geometry as per ASTM Standard (E8/E8M-13a), (**b**) Specimen prepared in two halves, (**c**) FSW tool geometry, (**d**) Fixture holding specimens, and (**e**) FSW welded specimens with supporting strips^42^.

Friction stir welding was established by customizing a computer numerically controlled (CNC) machining center to weld brass 405–20. A modifiable CNC program was developed by employing the spindle speed function and the feed function to vary both rotational and traverse speeds of the FSW tool. Nine empirical combinations were performed based on a full factorial method (refer to Table 2). Specimens were prepared in two splits, as shown in Fig. 1a,b, to be welded as per ASTM standard (E8/E8M-13a)^42^.

**Table 2:** L-9 DOE based on the full factorial method^42^.

DOE no	Rotational speed (revolutions per min)	Traverse speed (mm/ min)	Revolutionary pitch (revolutions per mm)
1	1600	60	26.7
2	1600	50	32.0
3	1600	40	40.0
4	1450	60	24.2
5	1450	50	29.0
6	1450	40	36.3
7	1300	60	21.7
8	1300	50	26.0
9	1300	40	32.5

The experimentation was carried out using Table 2, where three weld characteristics were evaluated namely, weld temperature, weld hardness, and weld strength. Moreover, all the welding output parameters/weld quality characteristics were found optimally interesting at the same FSW input parameters, i.e., friction stir welding factors (FSWFs). Table 3 summarizes the optimal weld conditions.

**Table 3:** Optimal welding conditions for three welding output parameters.

Sr. no.	Optimal FSWFs, levels		Weld quality characteristics
	TRS (RPM)	TTS (mm/min)	
1.	1450	60	Weld temperature (°C)
2.	1450	60	Weld hardness (HR)
3.	1450	60	Weld strength (MPa)

The specimen thickness was 5 mm, and the FSW tool’s immersion depth was 3.5 mm in all the FSW experiments for naval brass. Moreover, a few FSW parameters were kept fixed/constant. For example, the cylindrical FSW tool pin geometry was employed in the current work, which was fixed. And tool angle was also kept constant with respect to the top surface of the brass specimens to be welded. The tool axial force or tool clamping force was also constant during each FSW experiment; however, these forces were not measured.

The tool material selected for this study was molybdenum high-speed steel (M2 HSS). The chemical composition of M2 HSS typically includes 5–9.5% molybdenum, approximately 4% chromium, 1.5–6.5% tungsten, and minor additions of vanadium. The chemical composition of naval brass in terms of (Cu: Zn: Pb) can be found in Table 4.

**Table 4:** Chemical composition of naval brass.

Material	Cu	Zn	Pb	Sn
	Percentage (%)			
Naval brass 405 − 20	63.0	34.7	1.0	1.0

The welding tools were manufactured from M2 high-speed steel (HSS) rods utilizing precision lathe turning to obtain the designated shoulder and pin geometries, as illustrated in Fig. 1(c). The selection of M2 HSS was based on its superior hardness and excellent wear resistance at elevated temperatures, characteristics that are critical for the friction stir welding (FSW) of naval brass. Additionally, to ensure structural integrity under the substantial forces involved in welding, a specialized clamping fixture was engineered and produced using a machining center. The fixture, shown in Fig. 1(d), offers a rigid constraint along the X, Y, and Z axes, effectively reducing thermal expansion and mechanical displacement during tool operation. Furthermore, dedicated supporting strips were positioned at the interfaces of the workpieces (see Fig. 1(e)) to maintain the integrity of the weld morphology. These strips reinforce the specimen edges against distortions and flash formation induced by the joining process, thereby ensuring a stable and defect-free welding zone throughout the experimental trials.

## Data augmentation

**Fig. 2:**
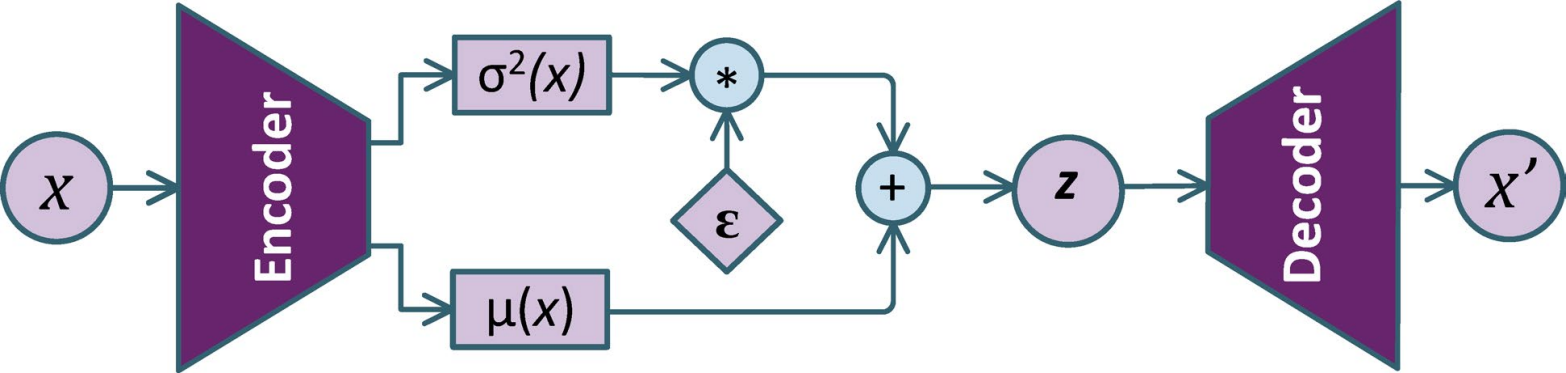
A diagram representing VAE architecture.

Variational autoencoders (VAEs) are used to encode input data by mapping it to a lower-dimensional latent space. Then, they use this compact representation to rebuild the original data. A fundamental characteristic of VAEs is the incorporation of a constraint that guarantees the latent space closely resembles a specified probability distribution^43^. In this framework, the latent space, which holds the most important parts of the data, is meant to follow a uniform distribution. The encoder component estimates the mean and variance of the latent variable (Z), while the decoder uses samples drawn from (Z) to reconstruct the original input, facilitating the generation of augmented data. The central aim of this study is to produce augmented data that aligns with the distributional characteristics of the training set by employing a standard VAE, as depicted in Fig. 2.

**Fig. 3:**
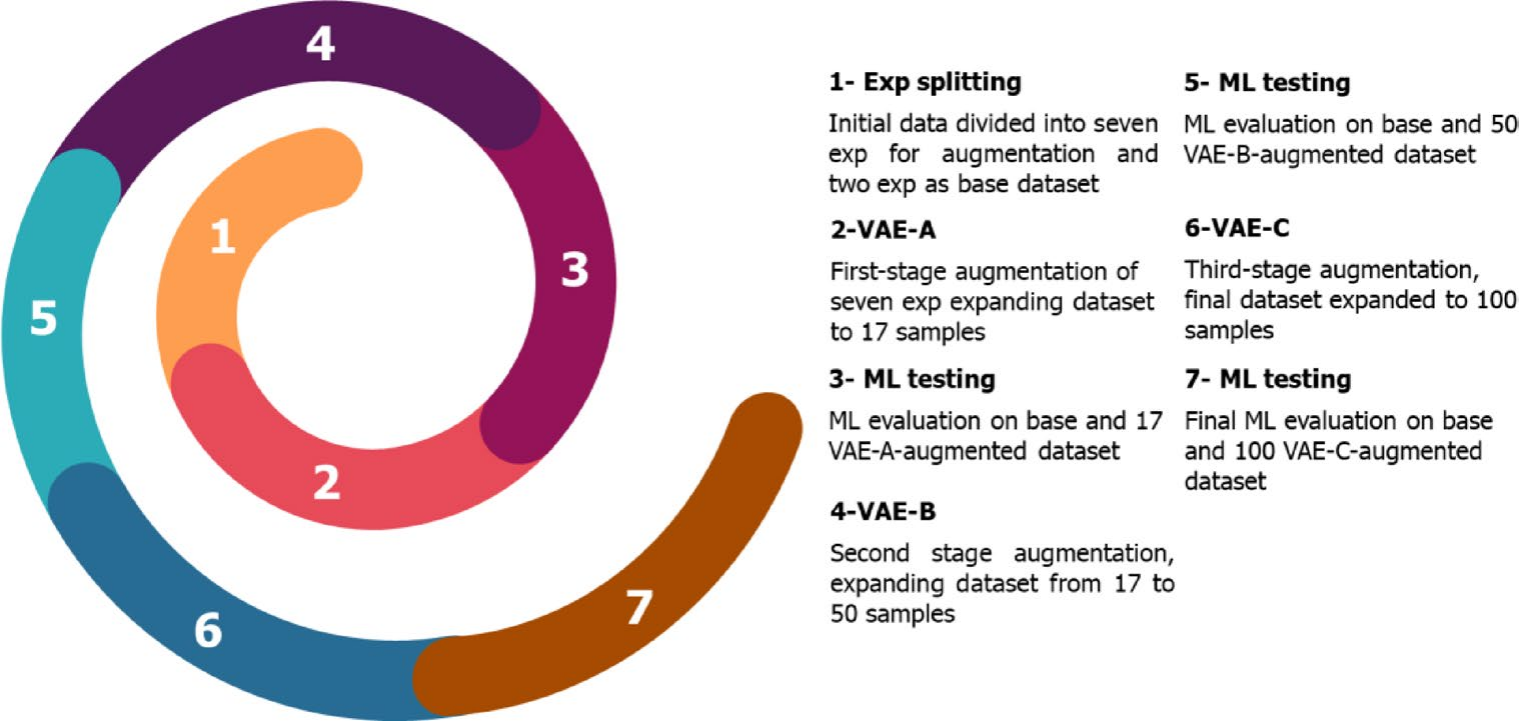
Schematic workflow of the VAE-based data augmentation and ML modeling strategy.

The methodology employed focused on iteratively improving a small dataset through VAE-based data augmentation to enhance the performance of ML models. There were nine experiments in the original dataset. It was split into two groups: one with seven experiments and the other with two. The smaller group, known as the base dataset, was only used for hold-out validation and not for data augmentation, model training, or testing. This approach ensured that the base dataset was exclusively used for validating the performance of each ML model, offering an unbiased assessment that was independent of the augmented data used during training and initial testing. As a result, the base dataset provided an objective gauge of the model’s generalization and robustness, ensuring that any observed improvements were not simply due to overfitting to the augmented data. The remaining seven experiments formed the initial dataset for augmentation. This foundational set underwent a series of augmentation phases, labeled as VAE-A, VAE-B, and VAE-C, in which the dataset size was progressively expanded. Specifically, VAE-A increased the dataset from seven to seventeen experiments, VAE-B further extended it to fifty, and VAE-C ultimately generated a dataset consisting of one hundred samples. This stepwise augmentation strategy (17, 50, 100) was employed to methodically assess the advantages of enlarging the data volume while minimizing the risk of significantly altering the original experimental data’s probability distribution. The objective of this process is to increase both the diversity and size of the training data, thereby supporting the creation of more robust and precise ML models. The full methodology for data augmentation and the implementation of ML models is depicted in Fig. 3.

During each augmentation phase (VAE-A, VAE-B, and VAE-C), the augmented dataset was first used to train and internally test the ML models using a standard training–testing split. Afterward, the models’ performance, trained on the augmented data from each phase, was evaluated by comparing their predictions to those of the base dataset. This stepwise approach allowed for a systematic assessment of how the progressive dataset expansion, generated by the VAE, influenced the predictive accuracy and generalization ability of the ML models when tested on both the internal test sets and the independent base dataset. This iterative process, progressing from VAE-A to VAE-C, is a strategic approach aimed at gradually expanding the dataset and evaluating its impact on the diversity of the synthetic data. By evaluating the performance of the ML models at each augmentation stage, the goal was to identify the optimal level of data augmentation that enhances model performance, while recognizing when further augmentation leads to diminishing returns or undesirable effects. This systematic approach allowed for an analysis of the trade-off between the benefits of additional synthetic data and the potential drawbacks, such as increased computational costs or the introduction of unrealistic or noisy data that could hinder model training^44^.

The datasets used in this study were formatted as comma-separated values files. All computational experiments were executed locally on a personal machine equipped with an NVIDIA RTX 3050 GPU, which significantly enhanced computational efficiency for ML tasks. Before model training, the input features were preprocessed using Min–Max scaling to ensure that all variables were represented on a comparable numerical range. For each feature *x* and *x’* were computed as:

1 $$\:{x}^{{\prime\:}}=\frac{x{-x}_{min}}{{x}_{max}{-x}_{min}}$$

where $\:{x}_{\mathrm{m}\mathrm{i}\mathrm{n}}$and $\:{x}_{\mathrm{m}\mathrm{a}\mathrm{x}}$denote the minimum and maximum values of that feature in the training set. This procedure rescales each feature to the interval [0,1]. Min–Max scaling was applied to the predictor variables used as model inputs, namely rotational and traverse speed. Four predictive models were evaluated in this study: SVR, XGBoost, AdaBoost and decision tree. Table 5 provides the hyperparameters used for these models. All four models were implemented in Python and treated as conventional machine-learning baselines to enable a consistent comparative assessment under the same experimental conditions.

**Table 5:** Hyperparameters for ML models.

Models	Parameter	Kernel/loss	Min	Max	Steps	Scale
SVR	C	-	0.1	1000	20	linear
	epsilon	-	0.01	10	20	linear
	kernel	rbf, linear, sigmoid	-	-	-	-
Adaboost	n_estimator	-	10	50	-	-
	learning rate	-	0.1	1.0	-	-
	loss	linear, square	-	-	-	-
XGBoost	n_estimator	-	70	100	10	linear
	max depth	-	4	6	1	linear
	learning rate	-	0.1	1	0.5	linear
DL	max depth	-	50	60	10	linear
	min sample split	-	30	40	-	-

## Results and discussion

In this study, predictive models were developed to evaluate key multi-parametric outcomes during friction stir welding of naval brass, specifically weld temperature, weld strength, and weld hardness. The findings significantly advance Industry 4.0 applications in FSW of naval brass by providing essential predictive insights that enable real-time monitoring and autonomous process optimization. The predictive capabilities of four ML algorithms DT, AdaBoost, XGBoost, and SVR were systematically assessed for their accuracy in forecasting these performance indicators. Model validation was conducted using three standard performance metrics: root mean square error (RMSE), mean absolute error (MAE), and mean absolute percentage error (MAPE).

To comprehensively assess the robustness and generalization ability of the ML models, a two-stage evaluation protocol was implemented. In the initial phase, the models were tested using a standard train-test split across each of the three incrementally expanded datasets: VAE-A, VAE-B, and VAE-C. The second phase entailed evaluating the trained models on data from two completely independent experiments, referred to as the base dataset, to yield critical information about their performance on out-of-distribution samples and overall generalization strength. The criteria for performance assessment RMSE, MAE, and MAPE, are formally defined in Eqs. 2–4.

2 $$RMSE = \sqrt {\frac{1}{N}\sum\limits_{{i = 1}}^{N} {(y_{i} - y_{i}^{ \wedge } )^{2} } }$$

3 $$MAE=\,\frac{1}{N}\sum\limits_{{i=1}}^{N} {|{y_i} - y_{i}^{ \wedge }|}$$

4 $$MAPE=\,\frac{1}{N}\sum\limits_{{i=1}}^{N} {|\frac{{{y_i} - y_{i}^{ \wedge }}}{{{y_i}}}|}$$

Where y_i_ and y^^^_i_ are actual and predicted values, N is the total number of samples.

### Validation of VAE-generated data

To ensure the reliability and representativeness of the virtual samples generated through the variational autoencoder, comprehensive validation was performed. The datasets generated (VAE-A, VAE-B, and VAE-C) were compared against the original data to assess their statistical similarity and structural consistency, for which a box chart with normal distribution plot and Pearson correlation heatmaps was employed to confirm that the synthetic samples preserve the underlying distributional characteristics of the real data.

**Fig. 4:**
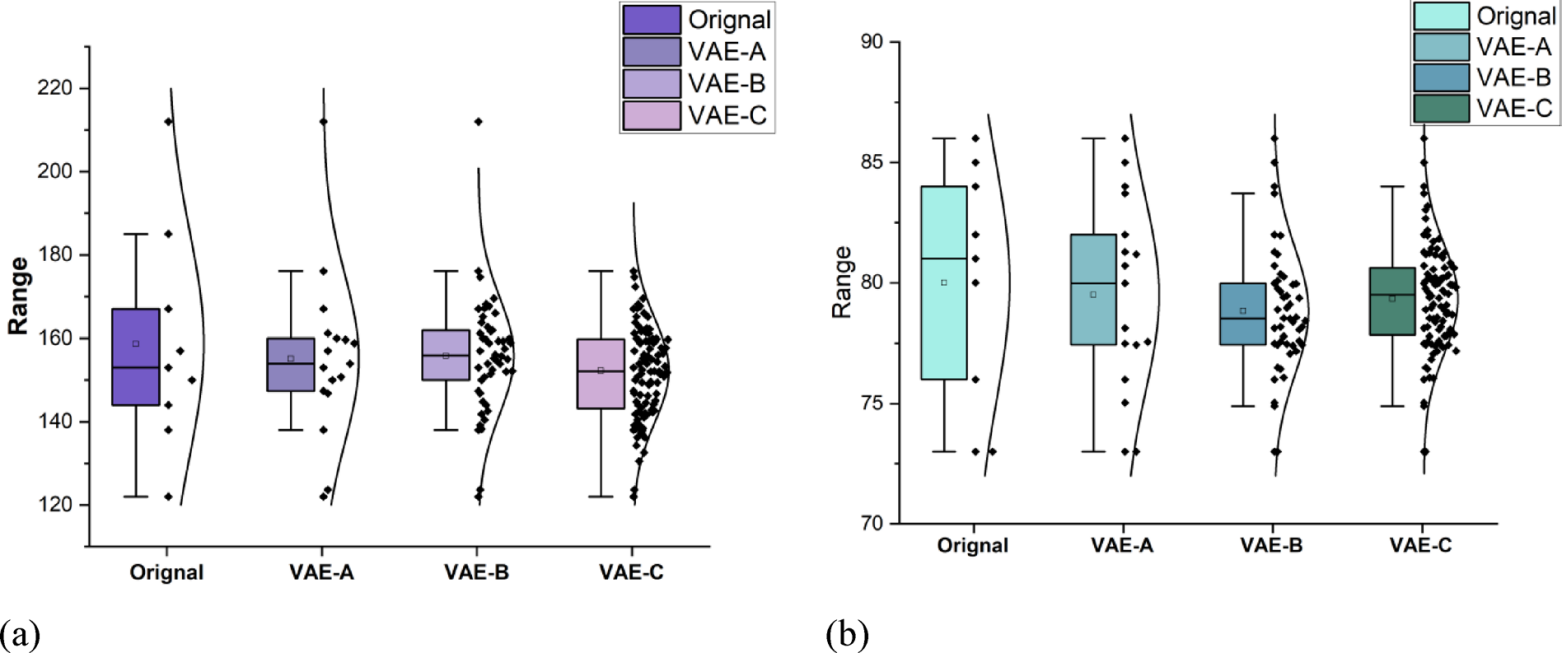
(**a**) Box chart with normal distribution plot for weld strength, (**b**) Box chart with normal distribution plot for weld hardness.

The analysis of the marginal distributions of key quality and process parameters, weld strength, weld hardness, and weld temperature, demonstrates the high fidelity of the VAE-generated data. By comparing the box-and-dispersion plots (as shown in Fig. 4) of the original data against the VAE-augmented versions (VAE-A, VAE-B, and VAE-C), it is evident that the VAE successfully learned the central tendency and dispersion of the features. Across all analyzed features, the median (central square) and the interquartile range (IQR) (the box itself, representing the middle 50% of the data) for the augmented datasets are nearly identical to the original data. The observed precision demonstrates that the VAE reliably reconstructs the most common and central regions of the feature space. For weld hardness, the models also produced data points corresponding to the extreme maximum outliers present in the original dataset. Preserving both the fundamental structure of the data and its extreme values is vital, as it ensures the augmented dataset encompasses the operational conditions. The uniformity observed across all analyzed marginal distributions further supports that the VAE is not simply replicating the training data but has effectively learned the underlying statistical mechanisms governing each feature.

**Fig. 5:**
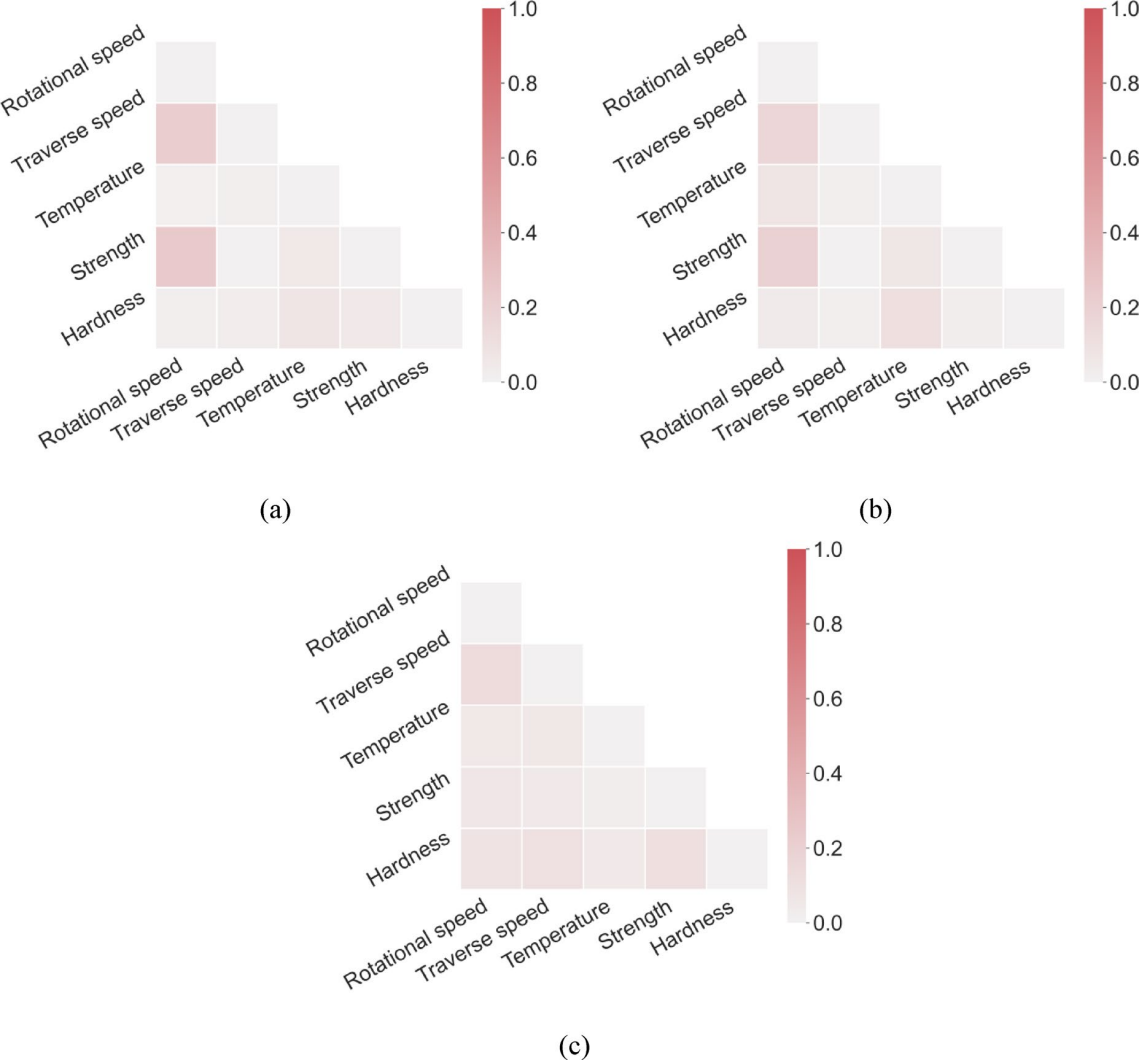
Heatmaps of the correlation (absolute divergence) of augmented data and the original data. (**a**) Original vs. VAE-A (**b**) Original vs. VAE-B (**c**) Original vs. VAE-C

While marginal distributions confirm individual feature fidelity, the multivariate structure of the linear relationships between variables must also be preserved for the synthetic data to be useful in a system context. This aspect was thoroughly evaluated by measuring the absolute divergence in the correlation matrices, defined as $\:|{\boldsymbol{R}}_{\boldsymbol{o}\boldsymbol{r}\boldsymbol{i}\boldsymbol{g}\boldsymbol{i}\boldsymbol{n}\boldsymbol{a}\boldsymbol{l}}-{\boldsymbol{R}}_{\boldsymbol{V}\boldsymbol{A}\boldsymbol{E}}|$. The resulting heatmaps of absolute divergence for VAE-A, VAE-B, and VAE-C (with greyish areas denoting zero difference) reveal a high degree of structural fidelity. Most correlation pairs exhibit values very close to zero (as illustrated in Fig. 5), signifying an almost exact correspondence with the original data. Importantly, the VAE models successfully captured and preserved the most significant original correlations, including those between speed and strength and between temperature and hardness. These associations are depicted as greyscale cells on the heatmaps, indicating that the VAE-generated data support analytical interpretations consistent with those derived from the original dataset. Minor divergences, highlighted in light red, are predominantly confined to correlations involving rotational speed (such as rotational speed versus traverse speed or strength). These deviations are minimal and typically occur when the original correlations were already weak (i.e., near zero). Overall, the consistently low absolute divergence throughout the correlation matrices demonstrates that the VAE has effectively captured and preserved the essential multivariate relationships, thereby affirming the structural validity of the augmented data.

### Predictive performance on augmented datasets

**Fig. 6:**
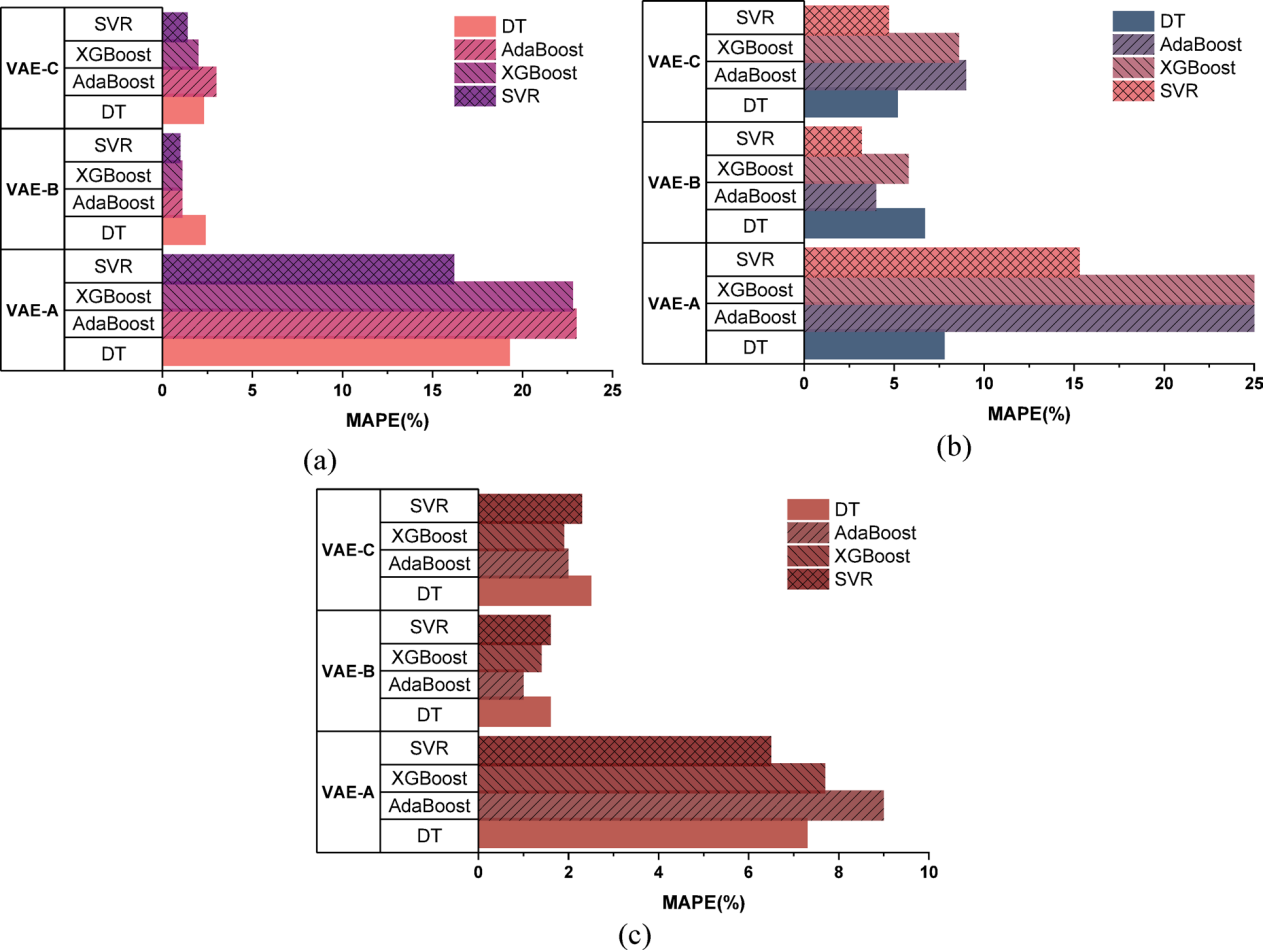
Performance of ML models using MAPE (**a**) weld temperature, (**b**) weld strength, (**c**) weld hardness.

For weld temperature prediction using the augmented datasets, model performance was assessed based on MAPE, as shown in Fig. 6(a), which highlights a distinct trend across varying dataset sizes. On the VAE-A dataset, all models exhibited relatively high MAPE values (e.g., AdaBoost at 23.0%, XGBoost at 22.8%, and SVR at 16.2%), reflecting reduced predictive accuracy with the smallest augmented set. In contrast, the VAE-B dataset yielded a marked enhancement in model performance, with AdaBoost (1.1%) and SVR (1.0%) attaining notably low error rates, and XGBoost closely matching at 1.1%. Notably, for the VAE-C dataset, an increase in MAPE was observed for AdaBoost (3.0%), XGBoost (2.0%), and SVR (1.4%), indicating that expanding the augmented dataset beyond 50 samples did not further enhance distributional accuracy for weld temperature prediction. Throughout all augmented dataset sizes, the decision tree model consistently exhibited higher MAPE values relative to the other three models.

For weld strength prediction using the augmented datasets, model performance was assessed through MAPE, revealing clear trends as dataset size varied. As with weld temperature, initial results on the VAE-A dataset indicated poor performance for AdaBoost (25.1%), XGBoost (25.0%), and SVR (15.3%), while the decision tree (DT) model exhibited relatively better accuracy at 7.8%, as illustrated in Fig. 6(b). Marked improvement was observed with the VAE-B dataset, where SVR achieved the highest accuracy (3.2%) and the lowest error among all models. AdaBoost also performed well at this stage (4.0%), whereas XGBoost (5.8%) lagged behind SVR and AdaBoost. In contrast, the transition to the VAE-C dataset led to increased MAPE for AdaBoost (9.0%), XGBoost (8.6%), and SVR (4.7%). DT, on the other hand, showed a gradual, though modest, reduction in MAPE from VAE-A (7.8%) to VAE-C (5.2%). Collectively, these findings underscore SVR’s optimal performance with the VAE-B dataset, while also highlighting diminishing improvements with further dataset expansion.

The weld hardness predictions on the augmented datasets are shown in Fig. 6(c); the MAPE evaluation showed a similar performance trajectory. On the VAE-A dataset, all models exhibited relatively high MAPE values (e.g., AdaBoost 9.0%, XGBoost 7.7%, SVR 6.5%). Performance significantly improved on the VAE-B dataset, where AdaBoost (1.0%) achieved the highest accuracy, closely followed by XGBoost (1.4%). SVR also showed strong competitive performance (1.6%) at this dataset size. However, consistent with observations for other parameters, a decrease in MAPE was noted on the VAE-C dataset for AdaBoost (2.0%), XGBoost (1.9%), and SVR (2.3%). DT’s performance for weld hardness was consistently less accurate compared to the other models across all augmented datasets. This suggests that for weld hardness, 50 augmented samples represented an optimal point for predictive accuracy for the top-performing models.

**Fig. 7:**
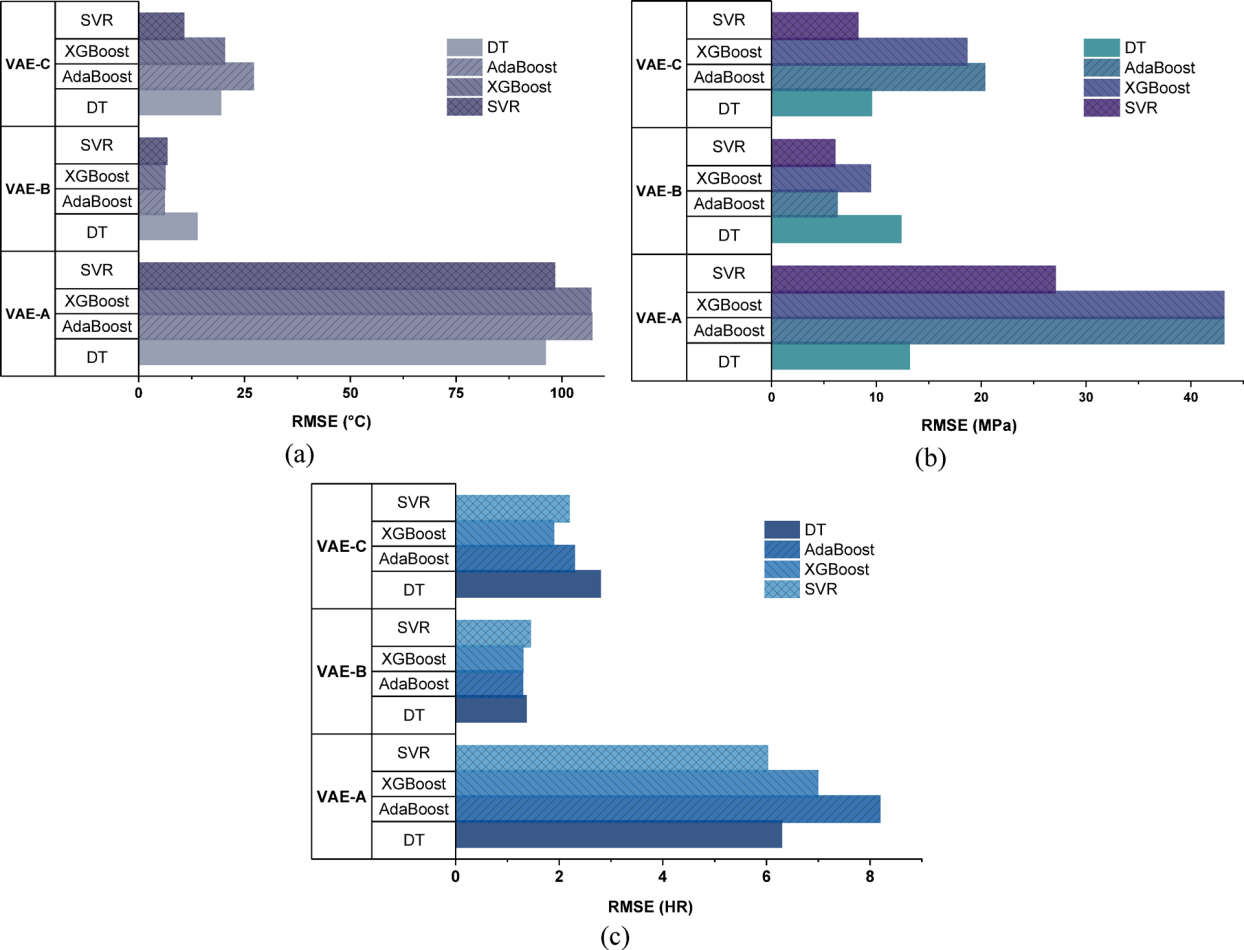
Performance of ML models using RMSE (**a**) weld temperature, (**b**) weld strength, (**c**) weld hardness.

As depicted in Fig. 7(a), the weld temperature prediction analysis based on RMSE indicates that the VAE-B dataset yielded the best accuracy. Under these conditions, AdaBoost achieved the lowest RMSE of 6.2 °C, with XGBoost and SVR exhibiting performance that was closely comparable. This represented a dramatic reduction from the exceptionally high RMSE values observed across all models on the VAE-A dataset, where errors frequently exceeded 100 °C, signifying substantial initial prediction inaccuracies. However, expanding to the VAE-C dataset led to a consistent increase in RMSE for AdaBoost, XGBoost, and SVR, suggesting that further augmentation did not enhance precision. Throughout these variations, DT consistently exhibited higher RMSE values compared to the other algorithms, indicating its less effective performance in predicting weld temperature.

Regarding weld strength prediction, Fig. 7(b) shows that the SVR model consistently performed best, as measured by RMSE. Specifically, it achieved a minimum RMSE of 6.1 MPa on the VAE-B dataset. This marked a significant improvement from its performance on the VAE-A dataset, where initial RMSE values were considerably higher. Ensemble models such as AdaBoost and XGBoost also yielded very high RMSE values on VAE-A, indicating poor initial fitting. While these models showed substantial improvement on VAE-B, their RMSE escalated again on the VAE-C dataset, suggesting a decline in predictive precision at the largest augmented scale. Conversely, DT displayed a distinct trend, demonstrating a gradual reduction in RMSE across all augmented datasets, from an initial 13.2 MPa on VAE-A down to 9.6 MPa on VAE-C, showcasing a more stable, even though generally less accurate, learning trajectory for weld strength.

Figure 7(c) details that for weld hardness prediction, AdaBoost and XGBoost jointly achieved the most precise RMSE at the optimal augmentation level, both registering an exceptionally low RMSE of 1.3 HR on the VAE-B dataset. This represented a sharp reduction from the comparatively high RMSE observed across all models on the VAE-A dataset. SVR also demonstrated strong RMSE performance on VAE-B, positioning it competitively among the top models. However, consistent with trends for other properties, moving to the VAE-C dataset generally resulted in an increased RMSE for AdaBoost, XGBoost, and SVR. This indicates that 50 augmented samples provided the most favorable conditions for minimizing RMSE in weld hardness prediction, with larger datasets proving less effective for sustained accuracy across most models.

The performance differences observed among the ML models can be largely attributed to their inherent algorithmic characteristics. SVR, for instance, proved highly effective in accurately estimating parameters like weld temperature and strength at optimal dataset sizes due to its ability to map complex, non-linear relationships by transforming data into higher-dimensional feature spaces, making it robust to intricate patterns. Conversely, the ensemble nature of AdaBoost and XGBoost, which sequentially builds upon the errors of preceding learners to produce a strong prediction, enabled them to capture underlying data structures adeptly. This characteristic was particularly beneficial for predicting the weld temperature and hardness, where these boosting algorithms excelled at optimal dataset sizes. However, specific sensitivities, such as AdaBoost’s susceptibility to noisy data or outliers, might explain instances of comparatively weaker performance, as observed with weld strength, and the general decline in accuracy for most models on the largest VAE-C dataset suggests potential overfitting or the introduction of less representative augmented data beyond a certain volume.

### Generalization and robustness on the independent base dataset

**Fig. 8:**
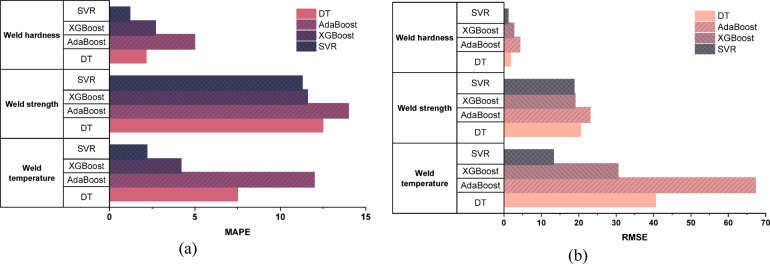
Generalization and robustness of ML models using (**a**) MAPE, (**b**) RMSE.

Analysis of model performance on the base dataset, as detailed in Fig. 8, reveals crucial insights into model generalization and robustness for real-world applications. While all models, as expected, showed relatively higher error metrics on this base dataset compared to their performance on the augmented datasets, SVR consistently demonstrated superior robustness and generalization. For weld temperature prediction, SVR demonstrated the most precise predictive capability, with considerably lower MAPE and RMSE values that were significantly more accurate than AdaBoost and XGBoost. A similar trend was observed for weld hardness, where SVR again recorded the lowest error metrics among all models. For weld hardness, while AdaBoost and XGBoost showed a slight performance advantage over SVR on the augmented datasets, SVR ultimately outperformed both models on the base dataset. This outcome particularly highlights the superior robustness of the SVR model. For weld strength, SVR’s performance was highly competitive, closely matching the accuracy of XGBoost while outperforming AdaBoost and DT. The SVR model’s consistent performance on an independent base dataset demonstrates strong generalization capability, indicating its reliability beyond the training data distribution. Such robust generalization is essential for practical deployment in Industry 4.0 environments, where models must operate effectively under varying real-world conditions.

While the models were also evaluated using MAE, these results were placed in a supplementary file to manage manuscript length. Consequently, the main text’s results primarily feature the MAPE and RMSE metrics. The detailed metric-based performance analysis across diverse weld characteristics illustrates that the models possess distinct strengths across different validation scenarios.

Despite these promising results, some limitations should be acknowledged. The proposed models were developed for naval brass under the investigated process conditions, so their applicability to other materials and welding settings remains to be validated. Future work should therefore focus on broader experimental validation and extension of the framework to other material systems and process conditions.

## Conclusion

This study highlights the effectiveness of robust ML predictive modeling for multi-parameter prediction in friction stir welding of naval brass. The main findings are as follows:

- The research demonstrates that ML predictive modeling can reliably predict weld temperature, weld strength, and weld hardness simultaneously, addressing a significant gap in predictive modeling for naval brass FSW.
- Among the algorithms evaluated, SVR consistently demonstrated the most robust and generalizable performance across both augmented and base datasets, achieving an MAPE of 1.0% for weld temperature, 3.2% for weld strength, and 1.6% for weld hardness on the best augmented dataset (VAE-B), demonstrating highly accurate predictions across all critical parameters.
- Data augmentation using a tailored variational autoencoder, coupled with rigorous validation, ensured the reliability and representativeness of the training datasets, overcoming the common challenge of limited experimental data.
- The predictive modeling developed in this study provides a conceptual foundation for smart, data-driven FSW process monitoring and control, enabling Industry 4.0 applications such as adaptive control, consistent weld quality, and reduced dependence on extensive physical trials.

## Supplementary Information

Below is the link to the electronic supplementary material.

Supplementary Material 1

## Data Availability

The experimental data supporting this study are included in the manuscript. Additional data may be made available by the corresponding author upon reasonable request.
