# Supplementary material for: Robust machine learning modeling for multi-parameter prediction in friction stir welding of naval brass: a case study towards industry 4.0
Source: Sci Rep. 2026 May 22;16:23432. doi: 10.1038/s41598-026-49005-0 (PMC13407878; doi:10.1038/s41598-026-49005-0)
Supplement: Supplementary file 1 — Supplementary Material 1 [file 41598_2026_49005_MOESM1_ESM.docx]

|  (a) |  (b) |
| --- | --- |
|  (c) | |

Figure S1: Performance of ML models using MAE (a) Weld temperature (b) Weld strength (c) Weld hardness

Figure S2: Generalization and robustness of ML models using MAE
